# Death after awakening from post-anoxic coma: the “Best CPC” project

**DOI:** 10.1186/s13054-019-2405-x

**Published:** 2019-04-03

**Authors:** Fabio Silvio Taccone, Janneke Horn, Christian Storm, Alain Cariou, Claudio Sandroni, Hans Friberg, Cornelia Astrid Hoedemaekers, Mauro Oddo

**Affiliations:** 1 Department of Intensive Care, Hopital Erasme, Université Libre de Bruxelles (ULB), Route de Lennik, 808, 1070 Brussels, Belgium; 2 Université Libre de Bruxelles, Brussels, Belgium; 3 Department of Intensive Care, Amsterdam UMC, University of Amsterdam, Amsterdam, The Netherlands; 4 Medical Department, Division of Nephrology and Internal Intensive Care Medicine, Charité-Universitätsmedizin Berlin, Berlin, Germany; 5 Intensive Care Unit, AP-HP, Cochin Hospital, Descartes University, Paris, France; 6 Department of Anaesthesiology and Intensive Care – Fondazione Policlinico Universitario Agostino Gemelli, Università Cattolica del Sacro Cuore, Rome, Italy; 7 Department of Anesthesiology and Intensive Care Medicine, Department of Clinical Sciences, Skåne University Hospital, Lund University, Lund, Sweden; 8 Department of Intensive Care, Radboud University Nijmegen Medical Centre, Nijmegen, Netherlands; 9 Department of Intensive Care Medicine, Centre Hospitalier Universitaire Vaudois (CHUV), University of Lausanne, Lausanne, Switzerland

**Keywords:** Outcome, Awakening, Cardiac arrest, Prognostication

## Abstract

**Background:**

In patients who recover consciousness after cardiac arrest (CA), a subsequent death from non-neurological causes may confound the assessment of long-term neurological outcome. We investigated the prevalence and causes of death after awakening (DAA) in a multicenter cohort of CA patients.

**Methods:**

Observational multicenter cohort study on patients resuscitated from CA in eight European intensive care units (ICUs) from January 2007 to December 2014. DAA during the hospital stay was extracted retrospectively from patient medical records. Demographics, comorbidities, initial CA characteristics, concomitant therapies, prognostic tests (clinical examination, electroencephalography (EEG), somatosensory evoked potentials (SSEPs)), and cause of death were identified.

**Results:**

From a total 4646 CA patients, 2478 (53%) died in-hospital, of whom 196 (4.2%; ranges 0.6–13.0%) had DAA. DAA was less frequent among out-of-hospital than in-hospital CA (82/2997 [2.7%] vs. 114/1649 [6.9%]; *p* < 0.001). Median times from CA to awakening and from awakening to death were 2 [1–5] and 9 [3–18] days, respectively. The main causes of DAA were multiple organ failure (*n* = 61), cardiogenic shock (*n* = 61), and re-arrest (*n* = 26). At day 3 from admission, results from EEG (*n* = 56) and SSEPs (*n* = 60) did not indicate poor outcome.

**Conclusions:**

In this large multicenter cohort, DAA was observed in 4.2% of non-survivors. Information on DAA is crucial since it may influence epidemiology and the design of future CA studies evaluating neuroprognostication and neuroprotection.

## Introduction

Despite continuous medical advances, the majority of patients who are initially resuscitated from cardiac arrest (CA) and admitted to the hospital die because of irreversible hypoxic-ischemic encephalopathy (HIE) [1, 2]. As such, research has focused predominantly on the implementation of potential neuroprotective strategies, like targeted temperature management (TTM) [3], as well as on the improvement of prognostication tools to early identify those patients with severe HIE [4, 5].

Around one third of deaths after CA are due to non-neurological causes, such as cardiogenic shock, sepsis, or multiple organ failure (MOF). Most of these deaths occur during the first 3 days after the return of spontaneous circulation (ROSC), which is earlier than HIE-related deaths [1]. However, delayed deaths from non-neurological causes, i.e., after TTM is discontinued, have been described [6]. Death after awakening (DAA) from post-anoxic coma may thus be misclassified as “neurological” death, since commonly used scores to assess long-term neurological recovery do not clearly distinguish between neurological and non-neurological causes of death. In one recent study, 16% of the CA patients who eventually died after ICU admission had recovered consciousness during their ICU stay [7]. Nevertheless, neurological assessment was limited, and no specific information on the results of additional prognostic tools was provided. This lack of consistency in outcome reporting after CA was also underlined by the Core Outcome Set for Cardiac Arrest (COSCA) initiative, which recently refined recommendations for how and when outcomes after CA should be measured and reported [8].

Thus, we developed this project called “Best CPC” (i.e., as for Cerebral Performance Category) with the aim to assess the prevalence of DAA in CA patients before hospital discharge. Moreover, we aimed to identify the characteristics of these patients, including predictors of neurological recovery, as we hypothesized that they might show no signs of poor outcome.

## Methods

### Study population

We retrospectively analyzed the institutional databases of eight academic ICUs where comatose CA survivors (i.e., a Glasgow Coma Scale < 9 at hospital admission) were admitted over the study period (January 2007 to December 2014). Among patients who died prior to hospital discharge, DAA was defined as a patient who was able to obey commands and open his/her eyes during the ICU stay but eventually died before hospital discharge for any reason, including a new neurological event. The local ethical committees in each country approved the study and waived the need for informed consent because of its retrospective nature.

### Patient management

All comatose patients were treated according to local protocols. Sedation policies, the site of temperature monitoring, and the specific findings on hemodynamic or ventilator management were not collected. Blood glucose levels were kept between 110 and 150 mg/dL using continuous intravenous insulin administration. A mean arterial pressure of at least 65–70 mmHg was maintained using volume resuscitation, dobutamine, and/or norepinephrine, whenever needed. Enteral nutrition was initiated according to local practice.

Withdrawal of life-sustaining therapies (WLST) was based on an interdisciplinary approach, involving intensivists and neurologists. Strong predictors of poor outcome were considered the bilateral absence of the N20 cortical responses to sensory evoked potentials (SSEPs) or persisting coma with poor motor response and absent brainstem reflexes at 3 days or more after CA. Less strong predictors (i.e., early status myoclonus, refractory status epilepticus, or elevated neuron-specific enolase (NSE) levels) were also considered, when available, in case of prolonged coma within a collegial agreement for WLST.

### Data collection

For each patient with DAA, demographics (including age, gender) and comorbid diseases (i.e., NYHA III-IV heart disease, COPD/asthma, diabetes, chronic renal failure requiring hemodialysis, liver cirrhosis, immunosuppression, previous neurological diseases such as cerebrovascular diseases, neurodegenerative diseases, or seizures) were recorded. Cardiac arrest variables according to the Utstein style (i.e., witnessed status, bystander CPR, location, time from arrest to ROSC, cardiac vs. non-cardiac causes of CA, initial rhythm) were collected [9, 10]. Time from arrest to neurological recovery as well as time from neurological recovery to death was recorded. Death was defined post hoc as “early” if it occurred within 9 days from arrest, according to the median time from arrest to awakening (see the “Results” section). Specific therapies during the ICU stay, including TTM (32–36 °C), vasopressors, inotropic agents, mechanical ventilation, renal replacement therapy (RRT), intra-aortic balloon pump counterpulsation (IABP), or extracorporeal membrane oxygenation (ECMO), were also collected. Overall ICU length of stay, the occurrence and site of infection, and presumed cause for death were reported for each patient.

Additional prognostic data, whenever available, included clinical examination (i.e., motor score of the Glasgow Coma Scale, with absent or posturing motor response defined as “poor”; absence of pupillary reflexes; myoclonus) and EEG (i.e., “highly malignant” EEG patterns [11], absence of reactivity to painful stimuli, seizures or status epilepticus) both on day 1 or at days 2–3 after arrest. EEG findings were collected from the daily report of neurologists in the medical record of each patient, and tracings were not re-analyzed. The presence of N20 cortical response of somato-sensory evoked potentials (SSEPs) was recorded as well as time from arrest to SSEPs. The highest NSE during the ICU stay was also collected. Results and timing of brain imaging (i.e., brain CT scan or magnetic resonance imaging (MRI)) were recorded.

### Statistical analysis

Statistical analyses were performed using IBM SPSS Statistics 24.0 for Windows. Descriptive statistics were computed for all study variables and normal distribution was assessed using the Kolmogorov–Smirnov test. Data are presented as count (percentage) or median [25th–75th percentiles]. Comparison between subgroups (IHCA vs. OHCA) was performed using the *χ*^2^ test or Mann-Whitney test, as appropriate. A *p* value < 0.05 was considered as significant.

## Results

A total of 4646 patients were admitted after successful resuscitation of a CA in the participating centers over the study period; of those, 2997 (64%) were OHCA and non-survivors were 2478 (53%). Main differences among centers are reported in Table 1.

**Table 1:** Main differences among participating centers. Data are presented as count (%) or median (ranges). The percentage of DAA is calculated on the total number of CA admissions

	Total (*n* = 4646)	OHCA/IHCA (*n* = 2997/*n* = 1649)	Non-survivors (*n* = 2478)	DAA (*n* = 196)	% of DAA	Time of awakening (days)
Center 1	778	762/16	515 (66%)	5	0.6	1 (1–1)
Center 2	384	244/140	207 (54%)	50	13.0	1 (1–42)
Center 3	408	211/187	235 (58%)	19	4.7	3 (1–9)
Center 4	393	274/118	237 (60%)	23	5.9	2 (1–7)
Center 5	598	420/178	284 (47%)	18	3.0	8 (3–44)
Center 6	311	273/38	148 (48%)	12	3.9	4 (2–8)
Center 7	1510	743/767	671 (44%)	45	3.0	2 (1–10)
Center 8	275	70/205	181 (66%)	24	8.7	5 (1–21)

*DAA* death after awakening, *OHCA* out-of-hospital cardiac arrest, *IHCA* in-hospital cardiac arrest

A total of 196 (4.2%) patients were identified among non-survivors as DAA; the proportion of these patients varied from 0.6 to 13.0% among centers. In 38 (19%) patients, DAA occurred after ICU discharge. The characteristics of the study cohort are shown in Table 2; 82 (42%) patients had an OHCA. DAA was less common among OHCA than among IHCA patients (82/2997 vs. 114/1649; *p* < 0.001). In the group of DAA patients after OHCA, heart failure, COPD/asthma, and the use of chronic hemodialysis, bystander CPR, and an initial non-shockable rhythm were less frequent than in the IHCA group. DAA patients from OHCA also had a longer time of ROSC, received more adrenaline, were more frequently treated with TTM, and experienced more shock than DAA patients after IHCA (Table 2).

**Table 2:** Characteristics of study population on admission and during the ICU stay. Data are presented as counts (percentage) or median [IQRs]

	All (*n* = 196)	OHCA (*n* = 82)	IHCA (*n* = 114)
Male, *n* (%)	132 (67)	60 (72)	72 (63)
Age, years	73 [62–79]	73 [65–79]	73 [60–79]
Estimated weight, kg	75 [70–85]	75 [70–82]	76 [70–89]
Comorbidities			
Chronic hypertension	75 (38)	29 (35)	46 (40)
Diabetes	60 (31)	24 (29)	36 (32)
NYHA III–IV heart failure	27 (14)	6 (7)	21 (18) *
Chronic coronary artery disease	86 (44)	32 (39)	54 (47)
Previous vascular neurological disease	32 (16)	18 (22)	14 (12)
Liver cirrhosis	17 (9)	4 (5)	13 (11)
COPD/asthma	50 (26)	14 (17)	36 (32) *
Chronic hemodialysis	30 (15)	7 (9)	23 (20) *
Immunosuppression	10 (5)	4 (5)	6 (5)
CA characteristics			
Time of ROSC, min	12 [6–20]	17 [12–25]	10 [5–15] *
Adrenaline, mg	2 [1–3]	3 [1–4]	1 [1–1] *
Witnessed, *n* (%)	167 (85)	66 (80)	101 (89)
Bystander CPR, *n* (%)	147 (75)	52 (63)	95 (83) *
Cardiac cause, *n* (%)	152 (78)	60 (73)	92 (81)
Non-shockable rhythm, *n* (%)	127 (65)	43 (52)	84 (74) *
ECPR, *n* (%)	15 (8)	5 (6)	10 (9)
After hospital admission			
Lactate on admission, mmol/dL	5.4 [2.9–8.4]	5.7 [2.9–8.4]	5.2 [2.9–8.4]
Vasopressor use, *n* (%)	178 (91)	74 (90)	104 (91)
Dobutamine use, *n* (%)	107 (55)	44 (54)	63 (55)
TTM, *n* (%)	117 (60)	66 (80)	51 (45) *
Infection, *n* (%)	89 (45)	36 (44)	53 (46)
Shock, *n* (%)	148 (76)	69 (84)	79 (69) *
IABP, *n* (%)	16 (8)	6 (7)	10 (9)
Post-ROSC ECMO, *n* (%)	13 (7)	3 (4)	10 (9)
RRT, *n* (%)	71 (36)	17 (21)	54 (47) *
MV, *n* (%)	195 (99)	82 (100)	113 (99)
Bleeding, *n* (%)^$^	48 (24)	16 (20)	32 (28)
ICU length of stay, days	8 [3–16]	9 [1–16]	7 [3–15]

*ECPR* extracorporeal cardiopulmonary resuscitation, *ECMO* extra-corporeal membrane oxygenation, *ICU* intensive care unit, *NYHA* New York Heart Association, *COPD* chronic pulmonary obstructive disease, *CA* cardiac arrest, *IABP* intra-aortic balloon counterpulsation, *TTM* targeted temperature management, *RRT* renal replacement therapy, *MV* mechanical ventilation

^$^Reduction of hemoglobin of at least 2 g/dL over 24 h requiring red blood cells transfusion

*p < 0.05 between IHCA and OHCA

**Fig. 1:**
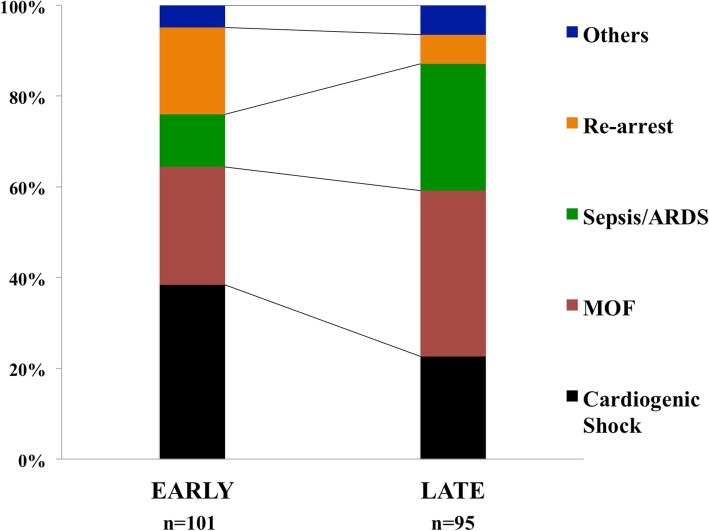
Distribution of different causes of death, according to early (≤ 9 days since neurological recovery) or late (> 9 days since neurological recovery) death

**Fig. 2:**
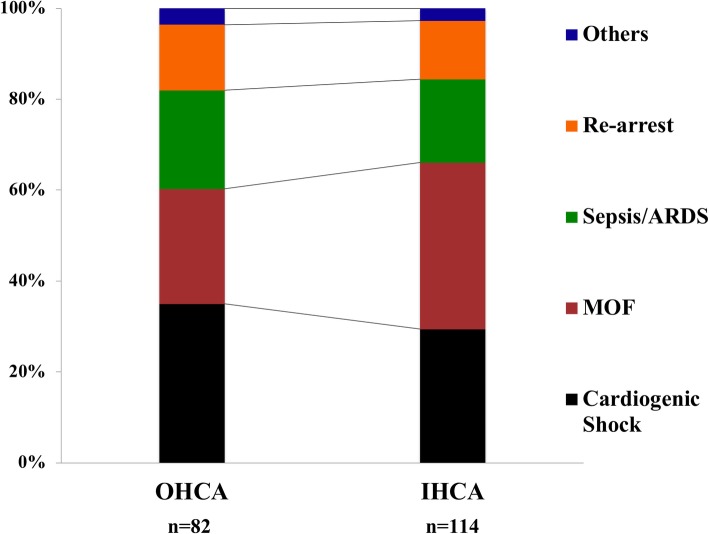
Distribution of different causes of death, according to in-hospital (IHCA) or out-of-hospital cardiac arrest (OHCA)

The median time from arrest to awakening was 2 [1–5] days; a large variability in time of awakening was observed among centers (Table 1). The time from arrest to awakening was similar in OHCA and IHCA patients (3 [1–5] vs. 2 [1–5] days; *p* = 0.77). The median time from awakening to death was 9 [3–18] days (9 [1–19] in OHCA vs. 9 [3–16] days in IHCA; *p* = 0.90). The causes of death in DAA were multiple organ failure (MOF) (*n* = 61), cardiogenic shock (*n* = 61), re-arrest (*n* = 26), severe acute respiratory distress syndrome (*n* = 20), sepsis/septic shock (*n* = 18), new diagnosis or ongoing cancer (*n* = 5), mesenteric ischemia (*n* = 3), and severe bleeding or pulmonary embolism (*n* = 1 each). The distribution of different causes of death showed that cardiogenic shock and re-arrest were more frequent among early non-survivors (*n* = 101), while sepsis/ARDS were more frequent among late non-survivors (Fig. 1; *p* = 0.008). The distribution of causes of death was similar between IHCA and OHCA patients (Fig. 2).

Main findings of clinical examination and additional prognostication tools in patients with DAA are reported in Table 3. On day 1 after admission, most patients (76%) presented a poor motor response and 38% had bilateral absence of pupillary reflexes. EEG was performed in only 30 (15%) patients; burst-suppression or suppressed EEG tracing was found in 3 (9%) patients, while most had a continuous (*n* = 21) or discontinuous (*n* = 6) background. Median NSE levels were 16 [15–27] μg/L, with only 1 patient exceeding 50 μg/L. A brain CT scan was performed in 52 (26%) patients; 2 abnormal CT findings were identified (subarachnoid hemorrhage and subdural hematoma). On day 3 after admission, clinical examination was available for 167 (85%) patients, because of some early deaths. Thirteen (8%) patients presented bilaterally absent pupillary reflexes. EEG was available in 56 patients, with 52 of them having a continuous and 4 of them a discontinuous background. NSE levels were 17 [14–27] μg/L, with only 1 patient exceeding 50 μg/L. Somato-sensory evoked potential were performed in 60 patients after a median of 4 [3, 4] days since the cardiac arrest; none of these patients had bilaterally absent cortical N20 responses.

**Table 3:** Prognostic findings of the study cohort. Data are presented as counts (percentage) or median [IQRs]

	Day 1		Days 2–3	
	*n*		*n*	
Motor response	196	1 [1–2]	167	5 [2–6]
M1–2 MR, *n* (%)	196	148 (76)	167	43 (26)
Bilateral absent PR, *n* (%)	196	74 (38)	160	13 (8)
Myoclonus, *n* (%)	196	3 (2)	167	0 (0)
Clinical seizures, *n* (%)	196	0 (0)	167	3 (2)
Continuous EEG, *n* (%)	30	21 (70)	56	52 (96)
BS/Suppressed EEG, *n* (%)	30	3 (9)	56	0 (0)
Reactive EEG, *n* (%)	26	22 (85)	44	40 (91)
NSE levels, μg/L	22	16 [15–27]	37	17 [14–27]
Abnormal brain CT-scan, *n* (%)	52	2 (4)	12	2 (16)
Bilaterally absent N20, *n* (%)	–	–	60	0 (0)

*M1–2* absent motor response or posturing, *MR* motor response, *PR* pupillary reflexes, *EEG* electroencephalography, *BS* burst suppression, *NSE* neuron-specific enolase, *CT* computed tomography, *N20* cortical response to somato-sensory evoked potentials on the median nerve

## Discussion

The results of this multicenter study showed that 4.2% of comatose CA survivors admitted to ICU die after regaining consciousness. We also observed large variability of DAA between participating centers. Most of these patients showed signs predicting good neurological outcome during the first 3 days after admission. Re-arrest was the most common cause of DAA in the early phase after CA while ARDS and sepsis were the most common causes of death thereafter.

Several studies have reported post-arrest shock, MOF, and other non-neurological conditions as the main causes of death in about one third of OHCA patients [2, 12, 13]. Nevertheless, no specific data on the occurrence of death among patients who eventually regained consciousness after the initial anoxic injury are available. In one study evaluating two different cooling strategies after OHCA [14], Deye et al. reported that a favorable neurological outcome was observed in 113 patients at day 90 after ROSC; 16 patients (4%), who initially had recovered neurological function, had died from non-cerebral causes at that time point. Also, Nobile et al. showed that, among 210 patients who died in the ICU after initial resuscitation from CA, 33 (16%) of them showed signs of improved neurological function, assessed by the Glasgow Coma Scale, prior to death. These patients presented more frequently with renal, cardiovascular, and respiratory failure than ICU survivors with good neurological function, and the authors concluded that extra-cerebral organ dysfunction may have resulted in their poor outcome [7]. Our multicenter study confirms that a proportion of non-survivors show neurological recovery prior to death before hospital discharge; this best neurological status of CA patients should be reported in all studies to better characterize the evolution of such patients, in particular if neuroprotective interventions are evaluated. In the recent COSCA recommendations on outcome reporting after CA [8], the need for early report on survival and neurological outcome was also underlined, although no specific definition was given. We used the term of DAA, although “Best CPC” (i.e., the lowest CPC score obtained during the entire ICU and hospital stay on a daily clinical assessment) or “CPC 6” (i.e., death for all causes after regaining consciousness in contrast with CPC 5, which would identify all non-survivors without regaining consciousness prior to death) could also be valuable alternative definitions of this finding.

Although non-cerebral causes of death have been described in earlier studies, we specifically reported their occurrence according to the timing from arrest to death. As expected, in the early phase, more than 60% of deaths were due to MOF and cardiogenic shock. Interestingly, 20% of patients with DAA died because of re-arrest. The incidence of re-arrest among patients immediately after ROSC can be as high as 80%, particularly in patients with shockable rhythms in the pre-hospital setting, although this might represent a non-sustained ROSC [15]. Also, while the incidence of re-arrest has been documented in the pre-hospital setting immediately after ROSC in up to 10% of patients [16], no previous reports have described the occurrence of re-arrest after hospital admission. We do not have enough data to conclude whether re-arrest in our cohort resulted from persistent circulatory failure, coronary lesion without effective revascularization, or if it was an unexpected complication (i.e., malignant arrhythmias) in hemodynamically stable patients. Among late deaths, 55% of them were still caused by MOF and cardiogenic shock and 26% by sepsis or ARDS while 3 patients had mesenteric ischemia. Sepsis-associated CA, in particular after IHCA, is a relatively common complication, which is associated with very low survival rates [17]. CA itself is considered a “sepsis-like” syndrome with a high degree of inflammation, organ dysfunction, and microcirculatory abnormalities that can significantly contribute to post-resuscitation mortality [18, 19]. In the TTM study, 500 out of 939 patients (53%) developed pneumonia, severe sepsis, or septic shock, which was associated with an increased mortality in a multivariate analysis [20]. In the Intensive Care Over Nations (ICON) database, 43% of all CA patients (*n* = 469) developed an infection during the ICU stay and 28% were in septic shock [8]; in the multivariable analysis, sepsis was also an independent predictor of ICU mortality. The occurrence of ARDS is a less common event after CA; 3 to 7% of patients presented this complication in a database including 812 resuscitated patients from 1998 to 2010 [21]. Nevertheless, this low rate might be due to the stringent definition criteria used in this study, as respiratory failure has been reported in up to 50–80% of CA patients admitted to the ICU [8, 22]. Finally, an intestinal injury is frequent after CA and is associated with the occurrence of endotoxemia, which might contribute to the worsening of post-resuscitation shock and the occurrence of organ failure [23]. However, the pathophysiology of this complication is poorly understood and its occurrence extremely rare [24]. As such, DAA occurs as the result of the ongoing disease or cardiac ischemia in the early phase after CA, while late deaths are usually due to the same causes as in non-CA ICU patients, i.e. infections and pulmonary problems. The presence of these potentially treatable causes of death, such as unexpected re-arrest, sepsis, or ARDS, underlines the importance of accurate monitoring and surveillance of CA survivors in the early phase. Future research should focus on the epidemiology of DAA in this setting, as well as the potential preventive and therapeutic measures to avoid these deaths (i.e., early coronary angiography, protective ventilation, digestive decontamination). Interestingly, in 19% of patients, DAA occurred after ICU discharge. This may suggest that those patients had shown full recovery of all acute dysfunction following CA and could be transferred to the ward; as such, DAA occurred for an unpredictable and new complication. Nevertheless, it is also possible that some limitations to an ICU re-admission could have been decided by ICU physicians; this decision may have resulted in death for a potentially treatable pathology that occurred in a patient with several comorbidities or a poor performance status.

The main impact of DAA concerns the evaluation of neuroprotective therapies and the assessment of neurological prognosis. Indeed, if a neuroprotective drug would show benefit and provide more patients recovering consciousness after the initial HIE, the assessment of neurological outcome few months after the initial event would be inadequate to demonstrate these effects, if patients die early from non-cerebral causes. Thus, DAA is a relevant “early” outcome to be reported together with long-term neurological outcome, quality of life, and cognitive function among CA survivors [8, 25]. In addition, if not properly addressed, DAA may cause false-positive results and reduce the prognostic accuracy of outcome predictors after CA [26–28]. We consider that future studies on prognostication tools and strategies should report the occurrence of DAA and exclude those patients who die in the very early phase after admission without a neurological examination, as they represent a source of potential bias for the interpretation of these results.

Some limitations of our study deserve discussion. First, the retrospective design might have produced an underestimation of DAA in these patients. Prospective, specifically designed studies will be needed to address this potential bias. However, should this be the case, it would mean that the incidence of DAA is even higher than that we observed in our study. Second, large variability in the description of DAA was observed; no specific analysis of factors associated with such variability was performed because of the lack of several confounders. This variability might be related to local medical practice (i.e., use of long-term sedatives might delay awakening and promote inappropriate WLST), limitations of care procedures (i.e., different timing for WLST might influence the possibility that some patients eventually awake later), or medical files quality. Third, awakening and obeying commands cannot be entirely considered as a “good neurologic outcome”; some patients with a CPC of 3 (i.e., conscious with severe cerebral disability) might present with the same definition used for DAA and still are categorized as “poor neurologic outcome” in all studies. Fourth, we did not collect data on non-survivors due to severe HIE or on survivors; these data might have provided additional comparisons on the reasons of death, time of death, and prognostication tools among groups. Fifth, prognostic indices were available only for a small proportion of these patients. This occurred because prognostication was not systematically performed in some participating ICUs and also because some patients awakened before prognostication, i.e., within the first 1–2 days after ROSC. Sixth, only the main cause of death was reported and no information on withdrawal or limitation of life-sustaining therapies decisions was collected. Also, we missed the clinical information on the pre-arrest condition of the patients, such as the Charlson comorbidity index, in particular for IHCA patients; this information might have influenced restrictions of life-sustaining therapies in some patients. The lack of specific ICU scores, such as the Acute Physiology And Chronic Health Evaluation (APACHE) II or Sequential Organ Failure Assessment (SOFA) scores, although not validated in the CA population, might also have been interesting to further characterize the severity of the study population. Seventh, the definition of “early” and “late” deaths was arbitrary and based on the median number of days from awakening to death and could not correspond to a clinically relevant time point. Finally, the lack of additional data on hemodynamics and ventilatory management limited further analyses on predictors of death and quality of care provided to such patients.

## Conclusions

4.2% of CA survivors admitted to ICU eventually die after regaining consciousness. This phenomenon has a large variability between reporting sites. These findings should be reported in neuroprognostication studies and may be helpful for the design of future trials assessing neuroprotective interventions.

## Abbreviations

ARDS: Acute respiratory distress syndrome
CA: Cardiac arrest
CPR: Cardiopulmonary resuscitation
DAA: Death after awakening
ECMO: Extracorporeal membrane oxygenation
EEG: Electroencephalography
HIE: Hypoxic ischemic encephalopathy
ICON: Intensive Care Over Nations
ICU: Intensive care unit
IHCA: In-hospital cardiac arrest
MOF: Multiple organ failure
MRI: Magnetic resonance imaging
NSE: Neuron-specific enolase
OHCA: Out-of-hospital cardiac arrest
ROSC: Return of spontaneous circulation
RRT: Renal replacement therapy
SOFA: Sequential Organ Failure Assessment
SSEP: Somato-sensory evoked potentials
TTM: Targeted temperature management
